# Grandchild care and life satisfaction of older adults: Empirical evidence from China

**DOI:** 10.3389/fpsyg.2023.1081559

**Published:** 2023-02-06

**Authors:** Xiangyu Dong, Hongxiang Ling, Tianyi Yang, Kun Wang

**Affiliations:** ^1^School of Humanities and Law, Zhejiang A&F University, Hangzhou, Zhejiang, China; ^2^School of Society and Psychology, Central University of Finance and Economics, Beijing, China; ^3^School of Philosophy, Zhongnan University of Economics and Law, Wuhan, China; ^4^Zhou Enlai School of Government, Nankai University, Tianjin, China

**Keywords:** grandchild care, older adults, life satisfaction, China, emotional support, subjective well-being

## Abstract

**Background:**

In China, grandchild care plays an important social role later in life. The effects of grandchild care on physical health and depression in older adults have been illustrated. However, there is a gap in research on grandchild care and life satisfaction of older adults specifically based on the Chinese experience.

**Method:**

Based on 7,079 individuals’ data from 2018 China Longitudinal Aging Social Survey (CLASS), this study explored the impact of grandchild care on older adults’ life satisfaction by using Ordinary Least Squares (OLS), Propensity Score Matching (PSM), and instrumental variables (IV) models.

**Results:**

The empirical results indicated that (1) life satisfaction was significantly higher for older adults who undertook grandchild care compared to those who did not; (2) non-coresiding grandparents showed higher life satisfaction than those non-carers, and this effect was not found in custodial grandparents or three-generation household grandparents; (3) higher life satisfaction of grandchild caregivers was achieved through reduced loneliness, enhanced self-efficacy, and increased emotional support from children, with the latter being the greatest contribution; and (4) the improving effect of grandchild care on life satisfaction was found mainly in the group of older adults who were male and in rural households.

**Conclusion:**

There was a significant difference in life satisfaction between older Chinese adults who provided grandchild care and those who did not. Efforts in terms of old age policy protection and family relationships should be made to enhance the subjective well-being of older adults.

## 1. Introduction

China has an aging population. The China Seventh Population Census shows that the number of older people aged 60 years and over has reached 264 million, accounting for 18.7% of the total population; the number of people aged 65 years and over is nearly 191 million, accounting for 13.5% of the total population^1^. Based on the above background, it is of profound theoretical and practical significance to improve the subjective well-being of older adults and use older workforces.^2^ In fact, a large proportion of Chinese older adults engage in paid or unpaid work, such as providing intergenerational care. Research has shown that more than half of middle-aged and older adults in China provide care for their grandchildren, a rate approximately 10 times that of South Korea (Ko and Hank, 2014). Hence, caring for grandchildren plays an important social role in the later years of Chinese individuals.

Why is grandchild care so common in China? In the traditional Chinese family setting, parents are morally responsible for the upbringing of their children and grandchildren, and the majority of families are now dual-income families with high work pressure and limited time and energy to raise their offspring. Older adults providing intergenerational care can increase their adult children’s education and employment opportunities, thereby improving overall family well-being (Burnette et al., 2013). Even as the labor force frequently shifts due to regional economic differences, leaving children behind has become common today (Chen and Liu, 2012). All of the above factors suggest that it is widely accepted that older people help their children care for their grandchildren in terms of traditional beliefs and practical needs. The proportion of grandparents coresiding with grandchildren is estimated to be as high as 45% (Chen et al., 2011). More than one-quarter of children in rural China have been reported as living only with a grandparent (Burnette et al., 2013). In urban China, taking care of grandchildren is a social participation with Chinese cultural character, one of the important family characters of older adults in China (Feng and Zhang, 2018).

In the traditional Chinese cultural context, the joy of family life is a true reflection of contentment in old age. Therefore, we are interested in whether grandchild care positively affects life satisfaction in older adults. From existing research, the impact of intergenerational care on the life satisfaction of older adults shows mixed results. There may be a positive, negative, or even no significant association between intergenerational care and the life satisfaction of older adults. A recent literature review suggests that the different forms of intergenerational care may be the main reason for this mixed outcome. In most studies, life satisfaction was higher for non-coresiding grandparents, lower for custodial grandparents and inconclusive for three-generation household grandparents when compared to non-carers (Danielsbacka et al., 2022). Meanwhile, based on data from 20 European countries, scholars have found that different cultural contexts also play an important role in the relationship between intergenerational care and life satisfaction. In countries where intergenerational care is socially expected, those who do not take on such a role are likely to experience lower life satisfaction (Arpino et al., 2018). Will this relationship be applicable to a Chinese culture that values family responsibilities and has a variety of caregiving forms? Unfortunately, few studies on grandchild care in China have focused on the areas of physical health and depression, and there is a gap in research regarding the influence of grandchild care on life satisfaction.

Theoretically, grandchild care may either positively or negatively impact the life satisfaction of older adults. The role strain theory suggests that grandchild care can cause older adults to assume multiple conflicting roles (Goode, 1960; Pearlin, 1989), thereby reducing life satisfaction. In contrast, role enhancement theory suggests that caring for grandchildren enhances the self-value of older adults (Sieber, 1974), which, in turn, increases their life satisfaction. Resolving this controversy requires empirical experience. In light of this, this study used OLS, PSM, and IV models to examine the effect of grandchild care on life satisfaction among older Chinese adults based on CLASS2018 data and to further analyze the mediating mechanisms and heterogeneity of this effect. To the best of our knowledge, this study is the first to examine the impact of grandchild care on life satisfaction in older Chinese adults.

The possible contributions of this study are as follows: First, we focus on the impact of grandchild care on the life satisfaction of Chinese older adults and explore how the traditional family nurturing model of grandchild care affects the life satisfaction of Chinese older adults. Most scholars have focused on the effects of grandchild care on the physical health and depression of older adults; few have focused on the effects of grandchild care on the life satisfaction of older adults. Second, the intrinsic mechanisms of grandchild care affecting older adults’ life satisfaction were systematically investigated, and the intrinsic mechanisms of grandchild care affecting life satisfaction were examined in terms of loneliness, self-value, and emotional support. The importance of different mechanisms was further examined using mechanism decomposition. Third, robustness estimation was performed using various estimation methods, such as OLS, PSM, and IV to guarantee the unbiasedness of the estimation results as much as possible.

## 2. Literature review and research hypotheses

There are various explanations for the formation of caregiving in scholarship. Existing research has been interpreted mainly on a benefit-seeking basis, suggesting that caregiving results from rational choices made by individuals after weighing up the benefits and costs. However, numerous studies have shown that caregiving does not bring significant benefits but can be a considerable burden. Parents do not feel a significant mood elevation when providing care (Kahneman et al., 2004), but providing care requires parents to sacrifice sleep, sex, and freedom. Given the explanatory impotence of the rational hypothesis, some scholars have proposed a new neurological explanation, suggesting that caregiving is the result of human evolution. This is typified by Brown et al.’s theory of selective investment, in which individuals with blood ties suppress their self-interest to promote the welfare of others in their time of need (Brown et al., 2011, p. 75–88). This biologically neurological characteristic also makes the impact of caregiving uncertain, as providing care is not the result of an individual’s rational choice.

In fact, there is no consensus among scholars regarding the impact of grandchild care on life satisfaction in older adults. One view maintains that grandchild care increases life satisfaction among older adults. According to the role enhancement theory, grandchild care leads to multiple roles, and older adults derive satisfaction from the role of the caregiver, which in turn increases life satisfaction (Sieber, 1974). Although the caregiving process may increase stress, the satisfaction derived from fulfilling multiple roles counteracts the risk of role strain and mitigates the negative effects of role stress. In terms of empirical results, some scholars have noted that older adults who provide support in raising grandchildren have better mental health and fewer conditions like depression (Tsai, 2016). From an intergenerational perspective, the provision of grandchild care by older adults is conducive to maintaining intergenerational relationships with their children and reducing the psychological burden of older adults, thus enhancing their psychological well-being (Bowers and Myers, 1999). Moreover, grandchild care can improve the lifestyle and dietary habits of older adults, contribute to the mental health of caregivers (Gattai and Musatti, 1999), and increase life satisfaction (Waldrop and Weber, 2001).

However, another strand of view argues that caring for grandchildren can reduce life satisfaction among older adults. From the perspective of the role strain theory, when individuals occupy multiple social roles, they face a series of competing role obligations, and this conflict is more pronounced when resources, energy, and emotions are limited (Goode, 1960). The process of weighing roles creates or increases role tension, which can become a “chronic stressor” harmful to health when the stress exceeds an individual’s capacity (Pearlin et al., 1990). Caring for grandchildren requires long hours of physical and emotional effort and also tends to breed negative emotions, such as tension and anxiety, which affect the mental health of older adults (Lumsdaine and Vermeer, 2015). Grandchild care causes a loss of energy in older adults, increases the workload of the family, contributes to worse mental health (Musil and Ahmad, 2002), and may even increase the level of depression in older adults (Hughes et al., 2007). In terms of the social participation dimension, grandchild care reduces the frequency of socialization of older adults and thus increases their mental burden (Jendrek, 1993). That is, caring for grandchildren can also become a burden for older adults and negatively impact their life satisfaction (Erbert and Alemán, 2008).

Indeed, we consider that the validity of these two theories may be related to the cultural context. In other words, the differences between role enhancement and role strain theory could stem from differences in individual perceptions of the appropriateness of multiple roles. When individuals take on multiple roles that are consistent with social norms, the applicability of role enhancement theory might be stronger and intergenerational caregivers have higher life satisfaction. Conversely, when individuals take on multiple roles that are not supported by social expectations, the applicability of role strain theory might be stronger and intergenerational caregivers have lower life satisfaction. For example, in countries where intergenerational care is socially expected, those who do not take on such a role are likely to experience lower life satisfaction (Arpino et al., 2018). In the Chinese cultural context, intergenerational care is a role that society expects grandparents to take on, and therefore, we believe that role enhancement theory is likely to be more applicable. In China, grandparents commonly provide grandchild care, which helps maintain their relationships with their families, promotes closer relationships between older adults and their adult children, and facilitates better social integration and participation in social activities through diverse social roles. At the same time, grandparents caring for their grandchildren can increase their adult children’s opportunities to participate in the labor market, increase family income, and improve the well-being of the entire family. Traditional Chinese culture places great importance on maintaining the productivity of older adults, encouraging them to maintain their self-value at a moral level by caring for grandchildren or taking care of household chores (Chen and Silverstein, 2000). Infused with Confucian ethics, grandchild care has become an obligation for older people because of blood ties. In terms of relationships with their children, caring for grandchildren is also an important way for older adults to participate in the lives of their children in exchange for their financial or emotional support. Based on the role enhancement theory and related literature, this study suggests that grandparents caring for grandchildren can improve older adults’ life satisfaction. Based on this and in the context of China’s unique traditional culture, we propose Hypothesis 1:

*Older adults who provide grandchild care have higher life satisfaction than those who do not.*

The differential association between intergenerational care and life satisfaction may stem from differences in care intensity. Coall and Hertwig argue that there is an inverted U-shaped relationship between intergenerational care and the health of older people (Coall and Hertwig, 2010, 2011). That is, the impact of intergenerational care on the well-being of older people may depend on caregiving intensity. Drawing on this perspective, Danielsbacka et al. (2022) categorized intergenerational care into three types: custodial grandparents, three-generation households and non-coresiding grandparents. They used this as a framework to review existing research, finding that in most cases, being custodial grandparents was negatively associated with older adults’ life satisfaction, being non-coresiding grandparents was positively associated with older adults’ life satisfaction, and living in three-generation households showed mixed results with older adults’ life satisfaction (Danielsbacka et al., 2022). The provision of intergenerational care is indeed a major challenge for older people in the context of limited strength (Baker and Silverstein, 2008). The stress created by excessive involvement in intergenerational care may counteract the life satisfaction-enhancing effects of intergenerational care for older adults. Therefore, we propose Hypothesis 2:

*The life satisfaction effect of intergenerational care is predominantly found in non-coresiding grandparents, while custodial grandparents and three-generation household grandparents do not have significantly higher life satisfaction than non-carers.*

Theoretically, caring for grandchildren may enhance life satisfaction in older adults through three pathways: alleviating loneliness, raising self-value, and receiving their children’s emotional support. According to the social emotional selection theory, aging leads older adults to prefer spending time with acquaintances and living in a relatively familiar social environment (Mather and Carstensen, 2005). As physical function declines with age, older adults’ social networks relatively atrophy (Van Tilburg, 1998), and loneliness becomes an important factor affecting older adults’ life satisfaction. The older one gets, the more one likes to maintain and strengthen ties with close social relationships (e.g., family and close friends) (Lang and Carstensen, 2002). This is when the family becomes a haven against loneliness in older adulthood. Grandchild care is an important way for older adults to participate in the lives of their offspring, through which they can gain the companionship of their children and grandchildren, thus enhancing their life satisfaction. Moreover, grandparent-grandchild interactions promote personal growth (Moore and Rosenthal, 2015).

From a personal value perspective, retirement can undermine the sense of self-worth of older adults; grandchild care gives them the opportunity to realize their self-worth. When adult children are too busy to care for their offspring, older adults can take on the work. This allows older adults to revalue themselves and develop a higher level of life satisfaction. Zhang and Chen found through their investigation that older Chinese adults can feel self-value in the intergenerational support they provide to their offspring, which is beneficial for their mental health status (Zhang and Chen, 2014). Providing grandchild care facilitates increased role-play, improves and expands the social integration and social scope of older adults, and enhances the sense of self-worth identity, which in turn contributes to increased physical and mental health (Szinovacz and Davey, 2006).

Emotional support from children is an important channel through which grandchild care affects life satisfaction among older adults. Older adults receive financial support and later-life care from their children through grandchild care, which is an intergenerational contractual relationship (Goodman and Silverstein, 2002). Along with the decline in work ability and income level, older adults can only take up household activities through childcare, which in turn reduces the opportunity cost of childcare for the offspring, achieving the optimal allocation of family resources, and retaining material resources and spiritual comfort from children (Croll, 2006). In terms of intergenerational exchanges, older adults who provide grandchild care receive emotional support from their children. That is, caring for grandchildren can strengthen the relationship between older adults and their children, bringing them closer. It has been found that for older adults, family support is more important than non-family support (Merz and Huxhold, 2010), and grandparent-child and grandchild relationships are both significantly and positively related to grandparent life satisfaction (Mahne and Huxhold, 2015). Xu used 2011–2013 CHARLS data and found that the health benefits of caring for grandchildren are primarily realized through emotional rewards (Xu, 2019). Therefore, we propose Hypothesis 3:

*The positive effect of grandchild care on older adults' life satisfaction is mainly achieved by reducing loneliness, enhancing self-efficacy, and increasing emotional support from children.*

The effects of grandchild care on life satisfaction may vary across contexts. From a gender perspective, a household division of labor within Chinese families, in which men earn money and women handle household chores, has been prevalent. Women have always taken on the role of family caregivers, and grandchild care in their later years has had a limited impact on life satisfaction. In contrast, older men are able to derive more mental comfort from grandchild care when they return to their families after retirement. This gender division leads to differences in the impact of grandfathers and grandmothers on their life satisfaction when providing intergenerational care. For example, Grundy et al. (2012) found that Chilean grandfathers involved in intergenerational care were more satisfied with their lives, but this effect was not found in grandmothers. More importantly, from the evolutionary perspective, women in their post-fertility era will help their daughters during their fertile period, known as the “grandmother effect” (Herndon, 2010). Therefore, the provision of intergenerational care by grandparents may stem more from biological instincts and have a limited impact on individual life satisfaction. From the perspective of household registration, there is a large out-migration of young laborers from rural China (Silverstein et al., 2006). Due to the absence of children, older rural adults emotionally need more companionship. Grandchild care can compensate for this deficit to some extent and increase life satisfaction. Moreover, grandparents provide care to children left behind and, in return receive remittances from their adult children (Cong and Silverstein, 2008). Therefore, we propose Hypothesis 4:

*The effect of grandchild care on improving life satisfaction was stronger for older male adults and rural households than for older female adults and those in urban households.*

## 3. Methodology

### 3.1. Data

**Figure 1:**
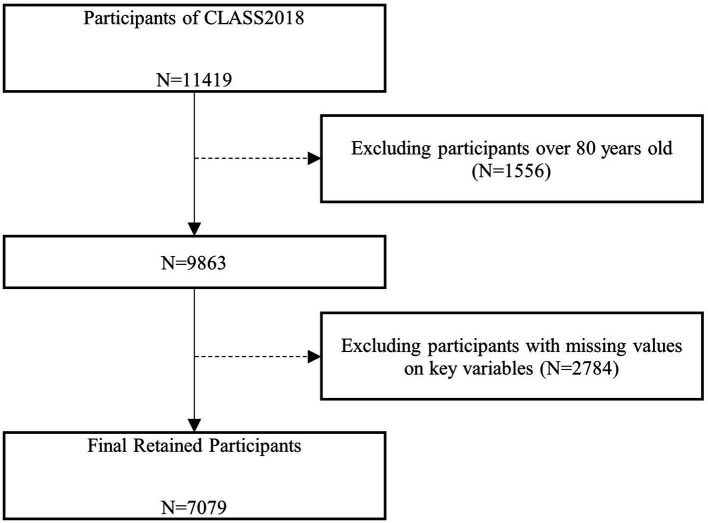
Data cleaning process.

The data used in this study were obtained from the 2018 wave of the China Longitudinal Aging Social Survey (CLASS), which was organized and implemented by Sun Yat-sen University. The survey adopted a stratified multi-stage sampling method with counties, county-level cities, and districts as primary sampling units, neighborhood committees and village committees as secondary sampling units, after which sample households in each of the sampled neighborhood committees and village committees, and one person aged 60 years or older in each sample household was selected as the survey subject. A total of 11,419 older individuals across 28 provinces were covered by CLASS2018, which is well-represented. According to the applicability of the research question, older adults aged 60–80 years were used as the study population. The two main reasons for excluding the over-80s sample are as follows. First, older adults over the age of 80 often do not take on the task of intergenerational care due to factors such as their health status and the fact that their grandchildren have reached adulthood. Second, those over 80 years old may have lower life satisfaction because of their health, and inclusion of them may lead us to overestimate the correlation between intergenerational caregiving and life satisfaction. Therefore, we excluded samples over 80 years old, and after further removing samples with missing and rejected responses on key variables on this basis, the valid sample size for this study was 7,079. The specific data-cleaning process is shown in Figure 1.

### 3.2. Variable measurement

#### 3.2.1. Dependent variable

The dependent variable was life satisfaction. This concept is understood as an individual’s assessment of their overall QoL (Diener et al., 1985). There are two ways of measuring this concept: one is based on a single question (Appleton and Song, 2008; Abbott et al., 2016) and the other is based on multiple questions (Wang et al., 2000). Most studies followed the first measurement, including Easterlin’s research on the relationship between economic growth and happiness in China (Easterlin et al., 2012). Cheung and Lucas found that a single-question measure of life satisfaction did not differ substantially from a multiple-question measure of life satisfaction (Cheung and Lucas, 2014). In this study, we used a single-question approach. Specifically, referring to Liu et al. (2020)’s study, this variable was obtained from the question “In general, how do you feel about your life satisfaction.” The respondents’ answers were divided into five categories: “very dissatisfied,” “relatively dissatisfied,” “average,” “relatively satisfied,” and “very satisfied.” Respondents’ life satisfaction was assigned a value from low to high, ranging from 1 to 5. A higher score indicates that the individual is more satisfied with their life. In addition, we added the sample over 80 years old and the missing data filled by the multiple interpolation method in the robustness test section respectively, and tested again based on the new sample to fully ensure the solidity of the empirical results.

#### 3.2.2. Independent variable

The independent variable in the study was grandchild care. This variable was obtained from the response to the question “In the past 12 months, did you provide care for your child’s children?” Referring to Yang and Yin’s (2022) study, we set this variable as a dummy variable. Older adults who had provided grandchild care in the past year were the subjects of interest in our study and were assigned a value of 1, while those who had not provided grandchild care in the past year were assigned a value of 0. In addition, considering that differences in the intensity of intergenerational care may affect the relationship between intergenerational care and older adults’ life satisfaction, a further distinction was made between types of intergenerational care based on existing research and the availability of data. Referring to Danielsbacka et al. (2022), this study categorized grandparents involved in intergenerational care into custodial grandparents, three-generation household grandparents, and non-coresiding grandparents. Specifically, based on the living arrangements of intergenerational carers, we considered those who live with their grandchildren but not with their children as custodial grandparents, those who live with their children and grandchildren as three-generation household grandparents, and those who do not live with their grandchildren as non-coresiding grandparents. Three types of intergenerational caregiver variables were generated, in each of which older adults providing the corresponding care were assigned a value of 1 and 0 otherwise.

#### 3.2.3. Control variables

Life satisfaction among older adults can be influenced by several factors. Referring to a previous study (He et al., 2022) and the availability of data, we controlled for both individual-and household-level variables. At the individual level, we controlled for respondent age (years), gender (male = 1, female = 0), marital status (married = 1, unmarried = 0), labor status (yes = 1, no = 0), education level (primary = 1, secondary = 2, high school and above = 3), health status (respondents’ self-assessed health, with 1–5 indicating very unhealthy – very healthy, respectively), and household registration type (respondents’ household registration, urban = 1, rural = 0). At the household level, we controlled for living arrangements (living with children = 1, otherwise = 0) and average monthly household expenditure (logarithmic transformed). In addition, we controlled for regional effects (eastern provinces = 1, non-eastern provinces = 0). In the robustness check section, we further considered physical pain (whether the respondent felt physical pain in the last month, yes = 1, no = 0), hospitalization status (whether the respondent was hospitalized in the previous 2 years, yes = 1, no = 0), and ADL (whether there was impairment in activities of daily living, yes = 1, no = 0).

#### 3.2.4. Mediating variables

Based on the previous analysis, this study chose to use loneliness, self-value, and children’s emotional support as mediating variables. Loneliness was obtained from the respondents’ perception of loneliness and is categorized as “not,” “sometimes,” and “often.” We took “not” as no loneliness with a value of 0, and “sometimes” and “often” as loneliness with a value of 1. Self-value was measured by the degree to which older adults agreed with the statement “I feel that I am still a useful person to society,” with a score of 1 to 5 indicating a range from “strongly disagree” to “strongly agree.” Emotional support from children was obtained from the emotional status of the older adults and their children and was categorized as “not very close” to “very close,” with a score of 1 to 5, respectively.

### 3.3. Model selection

Various methods were used in this study to accurately identify the association between intergenerational care and older adults’ life satisfaction. First, we briefly describe the sample data’s characteristics in the descriptive statistics section. Second, we tested the association between intergenerational caregiving and older adults’ life satisfaction using the OLS model in the benchmark regression section. We further analyzed the association between different types of intergenerational caregiving and older adults’ life satisfaction. Third, we re-estimated this relationship using the instrumental variables approach for the endogeneity problem raised by reverse causality and omitted variables. Fourth, in the robustness testing section, we applied PSM for possible self-selection effects and used a supplementary sample to test the effect of sample selection. Fifth, for the possible mediating channels, a non-parametric percentile Bootstrap method was employed, and the contribution of each channel was decomposed using the KHB method. Sixth, we used subgroup regressions to analyze heterogeneity in the impact of intergenerational care by gender and household registration.

#### 3.3.1. OLS model

Considering life satisfaction as a continuous variable, we first estimated the effect of caring for older adults on life satisfaction using an OLS model. The specific model settings are as follows:


$$Satisfaction_{i}=\alpha_{0}+\beta_{0}Care_{i}+\sum \gamma_{m}X_{mi}+\varepsilon_{i}$$

where $Satisfaction_{i}$ denotes the life satisfaction status of the ith older adult, $Care_{i}\;$

denotes whether the ith older adult provides grandchild care, $X_{mi}$ denotes other control variables, $\varepsilon_{i}$ denotes the random error term, $\beta_{0}$ is the coefficient to be estimated in this study, which reflects the magnitude and direction of the effect of grandchild care on the life satisfaction of older adults.

In addition, as the life satisfaction measure is scored on a scale of 1–5, it can also be interpreted as an ordinal variable and should be estimated using the orderd probit model. It has been shown that the sign and significance of the coefficients estimated by the OLS and orderd probit models are the same and that there is no difference between the OLS and orderd probit model estimates when the model is set up correctly (Ferrer-i-Carbonell and Frijters, 2004). Therefore, this study applied the OLS model for estimation and tested the validity of the results using the orderd probit model.

#### 3.3.2. IV model

The endogeneity of this study has two sources. One is the reverse causality. Older adults with higher or lower life satisfaction may be more inclined to provide grandchild care. The other is the omitted confounding variable. For example, older adults are more likely to provide intergenerational care when their children are in difficulty, but they may also have lower life satisfaction. To address this reverse causality problem, we use an instrumental variable approach for the estimation. Based on existing studies, we selected the number of grandchildren under the age of 18 years and the number of grandchildren living together among older adults as instrumental variables. Next, a two-stage least squares approach was used for the estimation, which consisted of two steps.

In the first stage, endogenous explanatory variables were regressed onto instrumental and exogenous explanatory variables.


$$Care_{i}=\alpha_{1}+\beta_{1}Z_{i}+\sum \gamma_{m}X_{mi}+\varepsilon_{i}$$

where $Care_{i}$ represents the grandchild care variable with endogeneity, $Z_{i}$ denotes the instrumental variable, $X_{mi}$ denotes the exogenous explanatory variable, and $\varepsilon_{i}$ is the random error term. The equation was designed to test the correlation between the instrumental and endogenous variables, and coefficient $\beta_{1}$ measured the degree of correlation between the two variables.

In the second stage, the fitted value of the first stage of $Care_{i}$ $\left(\overset{⌢}{Care_{i}}\right)$ was substituted into the OLS regression equation, which in turn yields a consistent estimate of *β2*, i.e., and derived the following:


$$Satisfaction_{i}=\alpha_{2}+\beta_{2}\overset{⌢}{\;Care_{i}}+\sum \gamma_{m}X_{mi}+\varepsilon_{i}$$

#### 3.3.3. PSM model

The caring behavior of older adults is not random but rather influenced by a variety of factors. This implies that there may be a self-selection effect on grandchild care and that differences in life satisfaction between grandchild caregivers and the ordinary older population arise from factors that influence the provision of caring for grandchildren rather than from the act of caring for grandchildren. For example, intergenerational caregivers in the United States are often found in low socio-economic status groups (Fuller-Thomson et al., 1997). This means that the impact of intergenerational care may come from selection effects rather than intergenerational care itself. Therefore, it is difficult to obtain unbiased estimates if we were to only use OLS regression. To address the non-random problem of grandchild care behavior, we used the propensity score matching method (PSM) proposed by Rosenbaum and Rubin (1983). The basic principle of PSM is to match individuals in the treatment group with individuals in the control group who are as similar as possible based on the probability of obtaining a treatment, thus achieving a randomized treatment. In this study, the treatment group comprised grandchild caregivers and the control group consisted of non-carers. The specific steps were as follows.

The first step is to use the logit regression model to calculate the propensity score:


PS(X)=Pr{D=1|X}=E{D|X}

where D is the dummy variable for whether the older adult was a caregiver. If the older adult was a caregiver, then *D* = 1; otherwise, *D* = 0. X represents the covariates that affect whether an older adult is a caregiver.

The second step is to match the treatment group with the control group based on propensity scores. The matching methods selected in this study were nearest neighbor matching, kernel matching, and radius matching.

The third step is to calculate the average treatment effect on the treatment group (ATT):


$$ATT=E\left(satisfaction_{1}|D=1\right)-E\left(satisfaction_{0}|D=0\right)$$

where $satisfaction_{1}$ represents the life satisfaction of the caregivers; $satisfaction_{0}$ represents the life satisfaction of the non-cares; and ATT is the difference between life satisfaction of the caregivers and life satisfaction of non-cares.

## 4. Empirical results

### 4.1. Descriptive statistics

Table 1 reports the results of the descriptive statistics. As can be seen, the proportion of older adults in the sample providing grandchild care was 29.1%, indicating that a significant number of older adults were involved in grandchild care. The mean value of life satisfaction for the sample was 3.820, indicating an overall high level of satisfaction among older adults. In terms of other characteristics of the sample, 50.9% were male, with a balanced gender ratio; the average age of the sample was 69 years, approximately 80% of the sample was married, 51% of the sample was registered in urban areas, and 32.6% were from eastern provinces.

**Table 1:** Descriptive statistics results.

Variable	Total			Grandchild caregivers	Non-carers	Difference (*t*-test)
	Obs	Mean	S.D.			
Dependent variable						
Satisfaction	7,079	3.820	0.821	3.885	3.794	0.091***
Independent variable						
Care	7,079	0.291	0.454			
Control variables						
Gender	7,079	0.509	0.499	0.515	0.507	0.008
Age	7,079	69.125	5.198	67.291	69.879	−2.588***
Marriage	7,079	0.753	0.431	0.801	0.734	0.068***
Education	7,079	2.144	0.785	2.211	2.116	0.095***
Work status	7,079	0.255	0.436	0.283	0.243	0.040***
Health	7,079	3.411	0.842	3.501	3.374	0.128***
Household registration	7,079	0.506	0.499	0.510	0.505	0.005
Living arrangement	7,079	0.268	0.443	0.341	0.238	0.103***
Household expenditure	7,079	7.359	1.004	7.507	7.299	0.208***
Province	7,079	0.326	0.469	0.339	0.321	0.018
Physical pain	7,079	0.547	1.129	0.548	0.546	0.002
Hospitalization status	7,079	0.258	0.438	0.246	0.263	−0.017
ADL	7,079	0.217	0.412	0.200	0.224	−0.024**
Mediating variables						
Loneliness	7,079	0.442	0.497	0.401	0.459	−0.058***
Self-value	7,079	2.978	1.078	3.069	2.941	0.128***
Children’s emotional support	7,079	1.992	1.247	2.00	1.988	0.013

** and *** indicate significance at the 5 and 1% levels, respectively.

**Figure 2:**
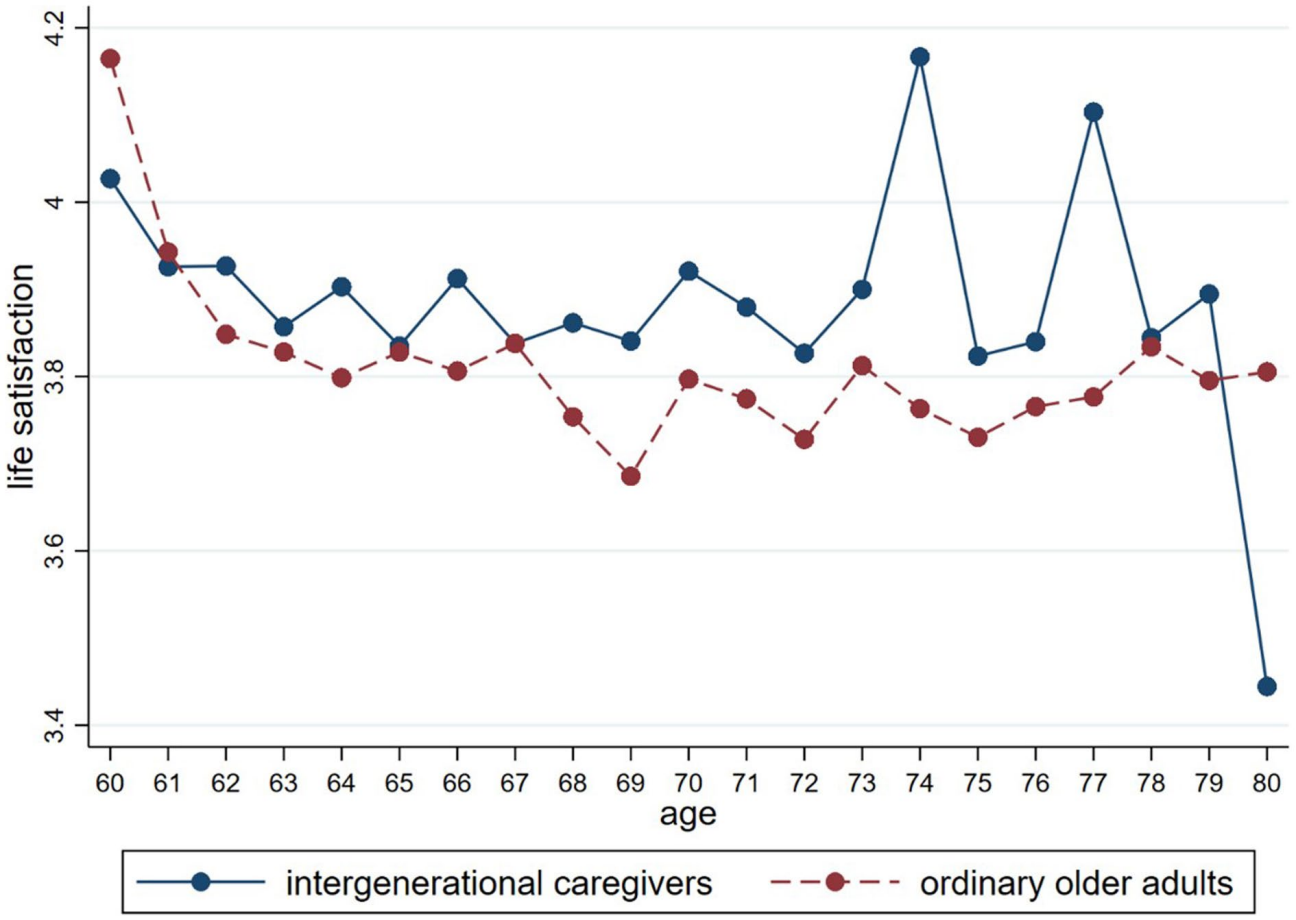
Comparative results by group.

In terms of the difference between older adults in grandchild care and non-carers, it can be seen that the life satisfaction of older adults who provided grandchild care was significantly higher than those who did not. We further compared the differences in mean life satisfaction between those providing grandchild care and the ordinary older population at different ages, and the results are shown in Figure 2. It was evident that older adults who provided grandchild care had a higher overall life satisfaction than non-carers, regardless of age. This finding was consistent with Hypothesis 1 and provided the basis for subsequent studies.

### 4.2. Benchmark regression

Table 2 presents the results of the benchmark regression analysis. Column (1) is the estimation result of the regression of the dependent variable on the core independent variables; column (2) is the estimation result after adding the control variables. It was not difficult to find that life satisfaction was 0.05 units higher for older adults who provided grandchild care compared to non-carers, and the results were significant at the 5% level. In addition, considering that life satisfaction is also an ordered categorical variable ranging from 1 to 5, we further estimated our results using the ordered probit model, and the results are shown in column (3), where caring for grandchildren remained significantly and positively associated with the life satisfaction of older adults. This indicated that older adults who provided grandchild care had higher life satisfaction than non-carers, tentatively supporting Hypothesis 1.

**Table 2:** Results of the benchmark regression.

	(1)	(2)	(3)	(4)
	OLS	OLS	Ordered probit	Different types
Care	0.091***	0.050**	0.069**	
	(0.021)	(0.021)	(0.029)	
Custodial grandparents				0.069
				(0.051)
Three-generation household grandparents				0.011
				(0.037)
Non-coresiding grandparents				0.063**
				(0.025)
Gender		0.020	0.033	0.020
		(0.019)	(0.027)	(0.019)
Age		0.002	0.002	0.002
		(0.002)	(0.003)	(0.002)
Marriage		0.061**	0.082**	0.062***
		(0.024)	(0.033)	(0.024)
Education		−0.005	0.000	−0.005
		(0.014)	(0.019)	(0.014)
Work status		0.050**	0.071**	0.050**
		(0.024)	(0.035)	(0.024)
Health		0.275***	0.380***	0.275***
		(0.013)	(0.019)	(0.013)
Household registration		0.079***	0.109***	0.079***
		(0.024)	(0.034)	(0.024)
Living arrangement		0.048**	0.068**	0.063**
		(0.022)	(0.032)	(0.026)
Household expenditure		−0.030***	−0.055***	−0.030***
		(0.012)	(0.017)	(0.012)
Province		0.298***	0.437***	0.297***
		(0.020)	(0.029)	(0.020)
_cons	3.794***	2.759***		2.764***
	(0.012)	(0.174)		(0.174)
N	7,079	7,079	7,079	7,079
r2/pr2	0.003	0.123	0.054	0.123

Robust standard errors are in parentheses. ** and *** indicate significance at the 5 and 1% levels, respectively.

We further analyzed the association between different patterns of intergenerational care and the life satisfaction of older people. As shown in column (4), using the non-carers as the reference, non-coresiding grandparents showed significantly higher life satisfaction, while both custodial grandparents and three-generation household grandparents showed higher, albeit non-significant, life satisfaction. This suggests that the positive association between intergenerational care and life satisfaction is mainly found among non-coresiding grandparents, proving Hypothesis 2 valid.

In terms of the estimated results for the other control variables, among the individual-level characteristics, having a spouse, self-rated good health, urban residence, and continuing to work were associated with higher life satisfaction among older adults. Among household-level characteristics, older adults who lived with children and had low household expenditures demonstrated higher life satisfaction than those who did not live with children and had high household expenditures. In addition, in terms of regions, older adults in the eastern provinces were happier than those in non-eastern provinces.

### 4.3. Endogeneity analysis

To address the endogeneity problem of the regression model, this study selected the number of grandchildren under 18 years of age and the number of grandchildren living together as instrumental variables for grandchild care and used two-stage least squares regression analysis (2SLS); the results are shown in Table 3. The results of the first-stage regression of the instrumental variables are shown in the column (2) of Table 3 with positive and significant regression coefficients, which is consistent with the expected results, indicating that the number of grandchildren under the age of 18 and the number of grandchildren living together were strongly correlated with the endogenous explanatory variable grandchild care. The F-statistic in the first stage was much greater than 10, which significantly excludes the problem of “weak instrumental variables.” The value of *p* of Hansen J statistic was 0.606, and the original hypothesis of “all instrumental variables are exogenous” could not be rejected, so the two instrumental variables were considered exogenous and not related to the random error term. This suggests that two instrumental variables – the number of grandchildren under the age of 18 and the number of co-resident grandchildren – were valid.

**Table 3:** Endogeneity analysis.

	(1)	(2)	(3)
	OLS	First stage	Second stage
Care	0.050**		0.150***
	(0.021)		(2.95)
Number of grandchildren under 18 years old		0.142***	
		(0.005)	
Number of grandchildren living together		0.126***	
		(0.011)	
F statistic		616.49	
Hansen J statistic		0.606	
N	7,079	7,079	7,079

Robust standard errors are in parentheses. ** and *** indicate significance at the 5 and 1% levels, respectively.

The regression results of the second stage showed that in column (3) of Table 3, the regression coefficients regarding the life satisfaction of older adults were significantly positive, and the effect of grandchild care on the life satisfaction of older adults was similar to the benchmark results estimated using OLS in the column (1), further validating the positive effect of grandchild care on the life satisfaction of older adults. However, in quantitative terms, the estimated coefficients of grandchild care increased in absolute value compared with the benchmark regression, suggesting that potential endogeneity led to an underestimation of the supportive effect of grandchild care on older adults’ life satisfaction.

### 4.4. Robustness check

These results suggest that older adults who provide grandchild care have higher life satisfaction than non-carers, but this finding needs to be further tested. Therefore, this study further used the PSM method to examine whether life satisfaction was higher among older adults who provided grandchild care than among those who did not, after eliminating selective bias. To make the PSM estimates more valid, we added variables such as physical pain status, hospitalization status, and ADL, to the original control variables that may affect the provision of grandchild care by older adults. A prerequisite to ensure the validity of PSM results was to pass the balance test. The results of the balance test are presented in Table 4. It can be found that the deviations of the matched treatment and control groups were less than 10% on all variables, proving that the balance test passed (Table 5).

**Table 4:** Balance test.

Variable	Unmatched	Mean		Bias%	Reduced	*t*-Value	Value of *p*
	Matched	Treated	Control		Bias%		
Gender	U	0.515	0.507	1.6		0.6	0.547
	M	0.515	0.529	−2.9	−83.4	−0.93	0.354
Age	U	67.291	69.879	−53		−19.54	0
	M	67.291	67.177	2.3	95.6	0.8	0.422
Marriage	U	0.801	0.734	16.1		6.01	0
	M	0.801	0.812	−2.6	83.6	−0.9	0.367
Education	U	2.211	2.116	12.1		4.61	0
	M	2.211	2.220	−1.1	91	−0.35	0.724
Work status	U	0.283	0.243	9.2		3.55	0
	M	0.283	0.273	2.2	75.7	0.7	0.482
Health	U	3.502	3.374	15.2		5.8	0
	M	3.502	3.479	2.7	82.3	0.87	0.382
Household registration	U	0.510	0.505	1		0.37	0.713
	M	0.510	0.519	−1.9	−95.4	−0.6	0.546
Living arrangement	U	0.341	0.238	22.9		8.94	0
	M	0.341	0.324	3.9	82.9	1.2	0.23
Household expenditure	U	7.507	7.299	21.2		7.94	0
	M	7.507	7.502	0.5	97.5	0.17	0.865
Province	U	0.339	0.321	3.8		1.44	0.149
	M	0.339	0.335	1	74.7	0.3	0.761
Physical pain status	U	0.549	0.546	0.2		0.07	0.942
	M	0.549	0.562	−1.2	−525.9	−0.35	0.724
Hospitalization status	U	0.246	0.263	−3.8		−1.46	0.144
	M	0.246	0.240	1.4	64.3	0.45	0.655
ADL	U	0.200	0.224	−6		−2.27	0.023
	M	0.200	0.201	−0.4	93.7	−0.12	0.902

**Table 5:** Average treatment effects.

Matching method	Treatment group	Control group	ATT	Bootstrap standard error	*t*-Value
Nearest neighbor match	3.885	3.835	0.050**	0.027	2.00
Kernel match	3.885	3.837	0.047**	0.019	2.12
Radius match	3.981	3.838	0.045**	0.020	2.00

** indicates significance at the 5% level; the standard error in row 5 is calculated by bootstrapping 500 times.

PSM usually requires the use of multiple matching methods to ensure the robustness of the results; therefore, this study used nearest-neighbor matching, kernel matching, and radius matching to calculate the average treatment effect. The results of the average treatment effects are reported. Among them, nearest neighbor matching, kernel matching, and radius matching indicated that the life satisfaction of older adults involved in grandchild care was 0.050, 0.047, and 0.045 units higher than that of those not involved in grandchild care, respectively. The results estimated using the three matching methods were very similar and remained consistent with the original conclusions. This indicates that Hypothesis 1 was supported.

In addition, the sample was supplemented to rule out the effect of excluding part of the sample in the estimation results. First, we re-estimated the relationship between intergenerational caregiving and life satisfaction based on 9,863 samples after filling in the missing values using the multiple interpolation method. Second, we included those over the age of 80 in the sample to explore the research questions based on all samples aged 60 years and over. The regression results are shown in Table 6. Column (1) shows the results after filling in the missing values, and column (2) shows the results after adding the sample over 80 years of age. We can observe that intergenerational care was significantly and positively associated with life satisfaction among older adults, regardless of whether the missing values were filled or those over the age of 80 years were included.

**Table 6:** Regression results of filled samples.

	Filling in missing values	Add to the 80+ sample
Care	0.053***	0.063***
	(0.019)	(0.020)
Control variables	yes	yes
N	9,863	8,680
R2	0.121	0.121

Robust standard errors are in parentheses. *** indicates significance at the 1% level.

### 4.5. Mechanism analysis

To address possible reverse causality in the mediation analysis, we used the fitted values of the independent variables at one stage in the two-stage least squares method to test for mediation effects. On this basis, we applied the nonparametric percentile Bootstrap method to test the mechanism of the effect of grandchild care on the life satisfaction of older adults (Bootstrap resampling number = 500, confidence level = 95%); if the 95% confidence interval of the mediating effect did not include 0, it indicated a significant mediating effect, and the test results are shown in Table 7. From the results, it was clear that the mediating effects of loneliness, self-value, and emotional support with children passed the 5% significance test, indicating that the mediating effects of these three variables were significant. The mediated effect sizes for loneliness, self-value, and children’s emotional support were 0.015, 0.009, and 0.070, respectively. This suggests that when older adults participate in grandchild care, they can improve their life satisfaction through mechanisms that reduce loneliness, achieve self-efficacy, and improve emotional support with their children; thus, Hypothesis 2 was verified.

**Table 7:** Bootstrap mediated effects test results.

Mediating variables	Total effect	Coefficient	Bootstrap standard error	*z* Value	*p* Value	LLCI	ULCI	Significance of mediation effect
Loneliness	Direct effect	0.135	0.046	2.36	0.003	0.025	0.217	Yes
	Indirect effect	0.015	0.006	2.35	0.019	0.002	0.028	
Self-value	Direct effect	0.142	0.051	2.80	0.005	0.031	0.241	Yes
	Indirect effect	0.009	0.004	2.16	0.031	0.001	0.017	
Children’s emotional support	Direct effect	0.080	0.051	1.58	0.113	0.002	0.222	Yes
	Indirect effect	0.070	0.013	5.47	0.000	0.047	0.097	

Next, we decompose the effects of the mediating variables using the KHB method. As shown in Table 8, children’s emotional support contributed nearly half of the total mediating effect (73.56%), while loneliness and self-value contributed 16.29 and 10.15%, respectively. This suggests that the link between grandchild care and older adults’ life satisfaction was achieved primarily through children’s emotional support, followed by feelings of loneliness, and was least influenced by self-value. In addition, the mediating effect of children’s emotional support accounted for 43.78% of the total effect, while feelings of loneliness and self-worth accounted for 9.7 and 6.04% respectively, with the sum of the three accounting for 60% of the total effect. This suggests that more than half of the impact of intergenerational care on older adults’ life satisfaction is due to these three mediating channels, illustrating to some extent the importance of the three mediating channels.

**Table 8:** KHB decomposition results.

	Loneliness	Self-value	Children’s emotional support
Effect size	0.015	0.009	0.066
Mediating rate	16.29%	10.15%	73.56%
Contribution to the total effect	9.70%	6.04%	43.78%

### 4.6. Heterogeneity analysis

Differences in satisfaction among older adults involved in grandchild care may be moderated by various factors. Therefore, this study further examined the differences in satisfaction between older adults involved in grandchild care and non-carers, moderated by gender and household registration factors.

The results of the heterogeneity analysis are presented in Table 9. In terms of gender, older male adults were significantly more satisfied with participating in grandchild care than with not participating in grandchild care, while older female adults did not differ significantly in their satisfaction with or without participating in grandchild care. In terms of household registration, caring for grandchildren did not have a significant effect on the life satisfaction of older adults with urban household registration, but for older adults with rural household registration, grandchild care significantly increased the life satisfaction of older adults. This proves that Hypothesis 3 holds.

**Table 9:** Heterogeneity analysis.

	By gender		By hukou	
	Male	Female	Urban	Rural
Care	0.059**	0.041	−0.007	0.100***
	(0.029)	(0.030)	(0.026)	(0.032)
Control variables	Yes	Yes	Yes	Yes
N	3,603	3,476	3,584	3,495
R2	0.134	0.114	0.160	0.120

Robust standard errors are in parentheses. ** and *** indicate significance at the 5 and 1% levels, respectively.

## 5. Discussion

Our empirical results show that grandchild caregivers have significantly higher life satisfaction than non-carers. This finding is consistent with Tang et al. (2022), who found, based on data from the China Health and Retirement Longitudinal Study, that grandchild caregivers had significantly lower levels of depression than those without grandchild care. Zeng et al. (2021) reported that older adults who provided grandchild care had fewer mobility limitations, fewer depressive symptoms, and better cognitive function. However, the findings of our study differed from those of other studies. A US study showed that grandparent caregivers among African Americans produced more depressive symptoms than grandparents who did not provide care, and more than 1/3 of caregivers and 1/5 of non-caregivers reported clinically relevant levels of depression (Fuller-Thomson and Minkler, 2000). From a gender perspective, grandmothers who provided grandchild care reported significantly lower self-satisfaction than those who did not provide grandchild care (Baydar and Brooks-Gunn, 1998). A possible reason for these differences is that motivations for grandchild care vary across cultural contexts (Hayslip and Kaminski, 2005). Older Chinese adults view grandchild care as a natural responsibility bestowed by blood ties, and they are happy to engage in such activities.

In terms of the different types of intergenerational care, we found that non-coresiding grandparents showed higher life satisfaction than those non-carers, and this effect was not found in custodial grandparents and three-generation household grandparents. This finding largely confirms the validity of Danielsbacka et al.’s classification of intergenerational care patterns. However, our findings are unique in that, unlike the majority of established research cases, which showed a negative association between custodial grandparenting and life satisfaction, our results showed higher but non-significant life satisfaction for those with custodial grandparenting. A possible reason for this difference is that Chinese older adults have internalized intergenerational care as their own responsibility (Xu, 2019), and although this pattern of caregiving has increased the burden on older adults, it has not reduced their life satisfaction. Moreover, intergenerational care in China is often caused by labor migration, especially in rural areas where young people go out to work and leave children behind (Cong and Silverstein, 2008). This differs from custodial parenting, represented by the United States, which is the result of parents caught in a difficult situation (Hayslip et al., 2019). As a result, Chinese custodial parents do not show significantly lower levels of life satisfaction.

In terms of its mediating effects, caring for grandchildren can enhance older adults’ life satisfaction by reducing their loneliness, increasing their self-value, and improving emotional support from their children. First, as older adults enter later life, their social networks inevitably shrink, and loneliness can seriously impair their quality of life. In terms of Chinese practice, older adults providing nurturing and companionship to their grandchildren can lead to a more fulfilling life (Yang and Yin, 2022), which in turn reduces their sense of loneliness. Based on Korean experiences, it has also been found that caring for grandchildren can predispose older adults to find meaning in their lives and perceive less stress (Park, 2018). Second, grandchild care enhanced the self-value of older adults. Some researchers have argued that intergenerational support reflects older adults’ self-value, which in turn enhances their life satisfaction (Carr and Gunderson, 2016). Our results further supported this hypothesis. Finally, we found that improved relationships with children were the predominant mediating pathway. Christiansen found that caring for grandchildren not only brought joy to grandparents but also strengthened their relationships and fostered a sense of closeness with their children (Christiansen, 2014). In contrast, less child–parent contact has been associated with increased depressive symptoms in parents (Buber and Engelhardt, 2008). Traditionally, due to the absence or inadequacy of the social welfare system, the vast majority of older Chinese adults solely receive social support primarily from their adult children (Cheng and Chan, 2006). This intergenerational support is one of the main sources of social support during the life course of a person, especially later in life, and plays an important role in a person’s physical and mental health (Zimmer and Kwong, 2003). Older adults enhance the emotional closeness between themselves and their children through grandchild care.

There were significant differences in the increase in life satisfaction obtained from grandchildren caring for different older age groups. On the one hand, older men experienced higher life satisfaction gains in grandchild care than female older adults. Based on data from the 2015 China Health and Retirement Longitudinal Study, Wang and Zhang (2021) found that providing grandchild care had a more pronounced positive effect on cognitive functioning in older men than in females (Wang and Zhang, 2021). On the other hand, from the perspective of household registration, rural older adults received higher life satisfaction from grandchild care than urban older adults. Similarly, Zhang and Liu (2022) found that grandchild care had a significant positive impact on the self-rated health of rural residents (Zhang and Liu, 2022).

There are three contributions of our study to the established theory:

First, our results confirmed the applicability of role enhancement theory in the Chinese context. Theoretically, the link between intergenerational care and the life satisfaction of older adults is inconclusive. Role enhancement theory supports positive associations (Sieber, 1974), while role strain theory supports negative associations (Goode, 1960; Pearlin, 1989). Our empirical results show that intergenerational caregivers have higher life satisfaction. This validates the applicability of role enhancement theory to the study of intergenerational caregiving. However, our findings do not entirely reject the role strain theory. After distinguishing among three different patterns of intensity in intergenerational care, the two higher-intensity intergenerational caregiver categories did not show significantly higher life satisfaction. This might suggest that the role of role strain theory is enhanced with higher intensity of care.

Secondly, our study examined the validity of the intergenerational care pattern classification. Earlier research on intergenerational caregiving often ignored differences in the intensity of intergenerational caregiving. The difference in the impact of intergenerational care often depends on the intensity of intergenerational care (Coall and Hertwig, 2010, 2011). Drawing on this perspective, Danielsbacka et al. (2022) categorized intergenerational care into three patterns: custodial grandparents, three-generation household grandparents and non-coresiding grandparents, and through a literature review found differences in the relationships between the three intensity types of care and the physical and mental health of older adults. Our study supports the validity of this classification to a certain extent. After categorizing intergenerational care into the three types mentioned above, we found that non-coresiding grandparents showed higher life satisfaction than non-carers. This effect was not found in custodial grandparents or three-generation household grandparents.

Third, our findings validate the selective investment theory and the grandmother effect to some extent. According to selective investment theory, individuals provide care because of social bonds, such as blood relations, rather than a choice based on weighing the benefits and costs (Brown et al., 2011, p. 75–88). The grandmother effect goes even further, stating that longevity after post-fertility is a uniquely human trait that contributes to grandmothers helping their daughters raise offspring (Herndon, 2010). In other words, it is likely that intergenerational care by grandmothers results from the logic of biological evolution and has little to do with life satisfaction. Our empirical results show that grandfathers providing intergenerational care exhibit higher life satisfaction, whereas this association does not appear for grandmothers. This empirical result somewhat supports the plausibility of the selective investment theory and the grandmother effect.

## 6. Limitation and strength

We acknowledge some limitations of this study. First, this study used cross-sectional data, which are correlational and cannot determine causality. Future studies could collect longitudinal data for cross-lag analyses and thus determine the direction of cause and effect. Second, although we have further divided intergenerational care into three types of varying intensity, this study almost only explored the effect of grandchild care on life satisfaction but not the intensity and quantity of care. In future studies, the effect of different intensities of care on the life satisfaction of older adults should be further investigated to gain a more comprehensive understanding of the effect of grandchild care on the life satisfaction of older adults. Third, the study was based on a unique Chinese context, which has some limitations in terms of external validity. China’s unique moral family relationships may be the cultural premise for the positive life satisfaction effect of grandchild care, and whether this effect holds in other cultures requires further discussion.

Beyond these limitations, our study still has some strengths. Firstly, we have placed our study in a uniquely Chinese context and are the first to examine the relationship between intergenerational care and life satisfaction in China. Secondly, we have employed a large sample for the study. China is a country of enormous size with large internal heterogeneity. Therefore, selecting representative data is a prerequisite for studying China’s issues. Contrary to existing studies based on a small number of Chinese provinces, our study selected a nationally representative sample of data, with a total sample size of 7,079. The conclusions obtained from the study broadly apply to the overall situation in China. Thirdly, we used a variety of robust estimation methods, including OLS, Oprobit, PSM, and IV estimation, to ensure the robustness of the estimates as much as possible.

## 7. Conclusion

It is not uncommon for older Chinese adults to provide grandchild care. This situation will become increasingly common, especially in the 4–2–1 nuclear family. Happiness in old age is the desired goal, and grandchild care is an important means for older adults to utilize their human capital. The study of the relationship between grandchild care and the life satisfaction of older adults has important implications for the promotion of both active aging and individual later-life arrangements. Based on CLASS2018 data, the present study explored this issue. The empirical results showed that older adults who provided grandchild care had higher life satisfaction than non-carers, and the results were significant at the 5% level. After classifying intergenerational care into three types: custodial grandparenting, three-generation household grandparenting and non-coresiding grandparenting, we found that the positive association with life satisfaction was mainly found in the non-coresiding grandparents. The mediated effects analysis revealed that loneliness, self-value, and children’s emotional support were the main ways in which caring for grandchildren enhanced their life satisfaction, with children’s emotional support contributing the most. The results of the heterogeneity analysis showed that the effect of grandchild care on life satisfaction was moderated by gender and household registration. Compared to females, urban household registration older adults, males, and rural household registration older adults were more able to enhance life satisfaction *via* grandchild care.

This study’s empirical findings have several important implications. First, when formulating delayed retirement policies and childbirth-friendly social security policies, the interplay between the two should be considered, and the grandchild-caring function of older adults should be fully developed. Although Article 2 of the Protection of the Rights and Interests of the Older Persons Law adopts the age limit of 60 in the definition of older adults, due to the improvement of social and medical security, 60-year-olds still have a positive impact on the value of their contribution to society; therefore, the best option needs to be chosen in the process of law-making and application of the actual situation in order to promote active aging. Although the national policy of delayed retirement is ostensibly beneficial to society’s development of older adult resources, it fails to take into account the important role of older adults in caring for grandchildren in the family and may be detrimental to family development. The benefits of delayed retirement may be offset by the burden placed on families by infant and child care. Second, local governments should actively provide fully functional and comfortable activities for older adults at low or no cost. This is also an important element emphasized in Chapter 4, Article 39 of the Protection of the Rights and Interests of the Older Persons Law, in terms of social services.^3^ Again, public policy support for family caregivers should take into account the perspectives of gender differences and urban–rural household registration differences, and special attention should be paid to the life satisfaction of older female and urban family caregivers. Finally, family development programs should be improved to enhance the sustainability of emotional and service support from adult children. Older adults caring for their grandchildren can reduce the stress in their children’s lives, and in return, provide emotional and service support to older adults, which creates favorable conditions for improving life satisfaction for all parties. Therefore, policymakers should further improve family policies to expand family care benefits through fiscal and tax policies to foster support systems for older adults and make child support sustainable.

## Data availability statement

The original contributions presented in the study are included in the article/Supplementary material, further inquiries can be directed to the corresponding author.

## Author contributions

KW: conceptualization, method, software, formal analysis, investigation, and resources. KW and TY: validation and data curation. XD: supervision, project administration, and funding acquisition. XD and TY: writing – review and editing. XD and HL: writing – original draft preparation. HL and TY: visualization. All authors have read and agreed to the published version of the manuscript.

## Funding

This research was supported by the Talent Startup Project Fund of Zhejiang A&F University (Program No. 2022FR022).

## Conflict of interest

The authors declare that the research was conducted in the absence of any commercial or financial relationships that could be construed as a potential conflict of interest.

## Publisher’s note

All claims expressed in this article are solely those of the authors and do not necessarily represent those of their affiliated organizations, or those of the publisher, the editors and the reviewers. Any product that may be evaluated in this article, or claim that may be made by its manufacturer, is not guaranteed or endorsed by the publisher.

## Supplementary material

The Supplementary material for this article can be found online at: https://www.frontiersin.org/articles/10.3389/fpsyg.2023.1081559/full#supplementary-material

## Footnotes

^1^www.stats.gov.cn/tjsj/tjgb/rkpcgb/qgrkpcgb/202106/t20210628_1818824.html.

^2^In this regard, the 2018 revision of the Law of the People’s Republic of China on the Protection of the Rights and Interests of the Older Persons, Article 8, paragraph 1, provides that “the State carries out education on the national situation of population aging and enhances the awareness of the whole society to actively deal with population aging.” It can be easily deduced that Chinese officials acknowledge the serious trend of population aging.

^3^People’s governments at all levels should be based on the level of economic development and demand for services for older adults, and gradually increase the investment in old-age services; people’s governments at all levels and relevant departments in the financial, tax, land, financing, among others must encourage and support enterprises, institutions, social organizations, or individuals to set up and operate day care for the older adults, older adult cultural and sports activities, and other facilities.
